# Can breeders prevent pathogen adaptation when selecting for increased resistance to infectious diseases?

**DOI:** 10.1186/s12711-022-00764-0

**Published:** 2022-11-08

**Authors:** Andries D. Hulst, Piter Bijma, Mart C. M. De Jong

**Affiliations:** 1 Quantitative Veterinary Epidemiology, Wageningen University & Research, P.O. Box 338, 6700AH Wageningen, The Netherlands; 2 Animal Breeding and Genomics, Wageningen University & Research, P.O. Box 338, 6700AH Wageningen, The Netherlands

## Abstract

**Background:**

Recent research shows that genetic selection has high potential to reduce the prevalence of infectious diseases in livestock. However, like all interventions that target infectious diseases, genetic selection of livestock can exert selection pressure on pathogen populations. Such selection on the pathogen may lead to escape strategies and reduce the effect of selection of livestock for disease resistance. Thus, to successfully breed livestock for lower disease prevalence, it is essential to develop strategies that prevent the invasion of pathogen mutants that escape host resistance. Here we investigate the conditions under which such “escape mutants” can replace wild-type pathogens in a closed livestock population using a mathematical model of disease transmission.

**Results:**

Assuming a single gene that confers sufficient resistance, results show that genetic selection for resistance in livestock typically leads to an “invasion window” within which an escape mutant of the pathogen can invade. The bounds of the invasion window are determined by the frequency of resistant hosts in the population. The lower bound occurs when the escape mutant has an advantage over the wild-type pathogen in the population. The upper bound occurs when local eradication of the pathogen is expected. The invasion window is smallest when host resistance is strong and when infection with the wild-type pathogen provides cross immunity to infection with the escape mutant.

**Conclusions:**

To minimise opportunities for pathogens to adapt, under the assumptions of our model, the aim of disease control through genetic selection should be to achieve herd-level eradication of the infection faster than the rate of emergence of escape mutants of the pathogen. Especially for microparasitic infections, this could be achieved by placing animals into herds according to their genetic resistance, such that these herds stay completely out of the invasion window. In contrast to classical breeding theory, our model suggests that multi-trait selection with gradual improvement of each trait of the breeding goal might not be the best strategy when resistance to infectious disease is part of the breeding goal. Temporally, combining genetic selection with other interventions helps to make the invasion window smaller, and thereby reduces the risk of invasion of escape mutants.

## Background

The possibility to use genetic selection as a strategy to combat infectious diseases in livestock has been recognized by animal breeders for a long time [1, 2]. In 1997, Bishop and Stear [3] showed that the potential to breed against nematode infections is greater than predicted by common quantitative genetic models. Recent theoretical work confirms the high potential of genetic selection to reduce infectious disease prevalence, that benefits from considerable indirect genetic effects in the transmission of infectious diseases [4–6]. An indirect genetic effect (IGE) is an effect of the genes of an individual on the phenotypes of other individuals [7–9]. For infectious diseases, these IGE arise because animals that are less likely to get infected are also less likely to infect other animals, simply because they are less often infected themselves. For this reason, genetic selection against infectious disease not only alters prevalence through the effect on an animal itself, but also through reduced exposure of its herd mates.

Apart from genetic selection, a great variety of strategies is and has been used in infectious disease control. Examples of such strategies are vaccination and treatment with antibiotic or antiviral drugs, but also hygienic and biosecurity measures, such as the separation of non-infected populations from infected ones using certification. These strategies have been used to successfully eradicate infections from populations; well-known examples are the eradication of Aujeszky’s disease [10, 11] and bovine tuberculosis (e.g. [12]) from many countries, and the global eradication of rinderpest [13, 14].

However, pathogens are not static, and any intervention strategy may exert selection pressure on the pathogen population. As long as an infection is not eradicated, this leads the pathogens to ‘adapt’ themselves to the intervention and, eventually, to evolve strategies to escape from it. The widespread antibiotic resistance is probably the most prominent example of this phenomenon, but escape from other interventions also occurs [15–17]. To prevent confusion between resistance of animals to an infection and resistance of pathogens to an intervention, we will refer to the latter as ‘escape’. So, pathogens may escape from the resistance of livestock to disease. When breeding livestock for lower infectious disease prevalence, it seems certainly possible that a pathogen will adapt itself to these selected animals and evolves escape from the genetic resistance of the animals. The recent identification of a new variant of infectious pancreatic necrosis (IPN) virus that causes relatively high mortality in genetically IPN-resistant Atlantic salmon illustrates this expectation [18]. In plant breeding, such examples of pathogen escape are widespread (e.g. [19]).

Escape of pathogens is a particular concern since genetic change in (micro-)pathogens is usually much faster than the genetic change that artificial selection can create in animals, because of high mutation rates, shorter generation times, and much larger population sizes (e.g. [20]). As noted by Bishop and Stear [21], the effects of selection for lower infectious disease prevalence might be diminished when pathogens get the chance to adapt themselves to the selected animals. Thus, to sustainably breed livestock for lower disease prevalence, it is essential to take the evolution of the pathogen into account and to try to prevent pathogen escape from occurring.

For some other interventions, strategies exist to prevent pathogens from developing escape. Therapies for Human Immunodeficiency Virus (HIV) that use a combination of several antiviral medicines at the same time is one example of such a strategy. An individual, mutated, pathogen strain might be able to escape from one or two of the used antivirals, but it is unlikely that it can escape from all, such that the combination of drugs kills all strains of the pathogen that are present in the host [17]. Another example is the restricted use of antibiotics. With this strategy, new antibiotics are used as little as possible and, when they are applied, a sufficiently high dose is used, such that all bacteria that are present in the host are killed. In this way the development of bacterial escape is prevented or at least largely delayed [22]. These examples illustrate that, to prevent pathogen escape, either no selection pressure should be applied or the selection pressure should be strong enough to fully kill the pathogen population in an individual or a group of individuals.

To our knowledge, strategies to prevent pathogen escape have not been investigated for artificial genetic selection of livestock populations against infectious diseases. Here, we investigate under which conditions escape mutants of a (micro-)pathogen can develop and spread through a closed livestock population. Specifically, we investigate how invasion of an escape mutant is influenced by the frequency and strength of the resistance of the host and the degree of escape of the pathogen. We develop a mathematical model of infection transmission that accounts for artificial genetic selection on a single resistance gene in the host population and for invasion of escape mutants from the pathogen population. We assume that the resistance gene only affects the propensity of an individual to become infected. Furthermore, for generality, we assume that, at any moment in time, a wide range of pathogen mutants can emerge. Hence, our focus is on the invasion risk of a new mutant that may emerge, rather than on evolutionary change in pathogen virulence, which has been investigated extensively elsewhere (see “Discussion”). Finally, we aim to identify strategies for genetic selection of livestock for lower infectious disease prevalence that limit or prevent the risk of pathogen escape.

## Methods and results

### Outline

In this section, we develop and analyse a mathematical model of infectious disease transmission that allows to investigate the conditions under which escape mutants of a pathogen can invade a host population that is under genetic selection for resistance to infection. The starting point of our analysis is a local, closed, host population (e.g., a herd of cattle without import of animals) that is endemically infected with a pathogen (the ‘wild-type’ pathogen). Hosts are then genetically selected for a single locus that confers some level of resistance to infection. Here, resistance merely implies that hosts are less likely to get infected with the wild-type pathogen, not necessarily that they cannot get infected at all. The aim of the selection is to reduce the prevalence of the infectious disease and, ultimately, to eradicate it from the local population.

Next, we assume that mutants of the pathogen that can to some degree escape host resistance can arise continuously as long as the wild-type pathogen is present in the host population. In other words, we assume that the escape pathogen is a mutant of the wild-type strain, so that it can arise only when the wild-type pathogen is (still) present. Our main interest is then to determine whether these escape mutants can invade the endemically infected host population and how the possibility to invade depends on characteristics of the host and of the pathogen, and on the degree of resistance against escape mutants provided by infection with the wild-type pathogen (cross resistance).

We start this section by describing the general epidemiological model that we use as the basis for our study. Next, we introduce genetic variation in the host population to allow for genetic selection for resistance in the host population. Here, we assume that host resistance is determined by a single bi-allelic locus, where resistance is either fully dominant or fully recessive. This model results in two host types: resistant hosts ($R$) and non-resistant hosts ($N$). Then we derive expressions for the prevalence of the pathogen in resistant and non-resistant hosts, and for the frequency of resistant hosts needed to eradicate the wild-type pathogen. Next, we further expand the model by allowing pathogen mutants to escape host resistance. Using this model, we assess how the possibility of the mutant to invade the host population depends on the frequency of resistant hosts, the level of resistance provided by the resistance gene, the fitness benefit of the escape mutant in resistant hosts, and the costs of the escape mutation for infection of non-resistant hosts. Finally, we incorporate infection of hosts with both types of pathogen at the same time into the model, to assess the effect of the degree of cross resistance on the possibility of the mutant to invade. To enhance comprehensibility of our complex model, we combined the methods and results sections in a fairly unusual way. The description of each step in the model, as outlined above, is followed by a short section with the results relevant to that step. Table 1 shows a notation key.

**Table 1:** Notation key

Symbol	Definition
$N$	Population size
${S}_{N}$	Number of non-resistant hosts in the susceptible state
${S}_{R}$	Number of resistant hosts in the susceptible state
${I}_{NW}$	Number of non-resistant hosts infected with the wild-type pathogen
${I}_{RW}$	Number of resistant hosts infected with the wild-type pathogen
${I}_{NE}$	Number of non-resistant hosts infected with the escape mutant pathogen
${I}_{RE}$	Number of resistant hosts infected with the escape mutant pathogen
${I}_{NWE}$	Number of non-resistant hosts infected with both pathogen types
${I}_{RWE}$	Number of resistant hosts infected with both pathogen types
${I}_{W}$	Total number of hosts infected with the wild-type pathogen (${I}_{NW}+{I}_{RW}+{I}_{NWE}+{I}_{RWE}$)
${I}_{E}$	Total number of hosts infected with the escape mutant pathogen (${I}_{NE}+{I}_{RE}+{I}_{NWE}+{I}_{RWE}$)
${f}_{R}$	Frequency of resistant hosts
$\beta$	Transmission rate parameter
$\alpha$	Recovery rate parameter ($\alpha =1$ is assumed throughout)
$r$	Cross resistance parameter ($r=1$ for full cross resistance, $r=0$ for no cross resistance)
${P}_{W}^{*}$	Equilibrium prevalence of wild-type pathogen ($({I}_{NW}^{*}+{I}_{RW}^{*})/N$)
${P}_{NW}^{*}$	Equilibrium prevalence of wild-type pathogen in non-resistant hosts (${I}_{NW}^{*}/N$)
${P}_{RW}^{*}$	Equilibrium prevalence of wild-type pathogen in resistant hosts (${I}_{RW}^{*}/N$)
${R}_{NW}$	Basic reproduction ratio for wild-type pathogen in non-resistant hosts
${R}_{RW}$	Basic reproduction ratio for wild-type pathogen in resistant hosts
${R}_{NE}$	Basic reproduction ratio for escape mutant pathogen in non-resistant hosts
${R}_{RE}$	Basic reproduction ratio for escape mutant pathogen in resistant hosts
${R}_{INV}$	Reproduction ratio of invasion of escape mutant in population endemically infected with wild-type pathogen

### SIS-model

Because the purpose of this study was to investigate the basic conditions under which pathogens can escape genetic resistance of hosts, without getting lost in mathematical details of specific cases, we decided to use the relatively simple, but very well-established Susceptible-Infectious-Susceptible (SIS) epidemiological model. This model provides a realistic representation of the transmission of several endemic infectious diseases [23], also in livestock populations.

**Fig. 1:**
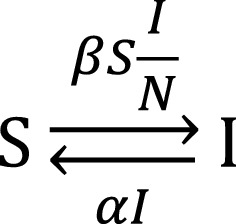
Susceptible-infectious-susceptible model

In the SIS-model, host individuals can be in the susceptible ($\mathrm{S}$) or the infected ($\mathrm{I}$) state (Fig. 1). Infected individuals are also infectious, meaning that they can infect susceptible individuals. In the context of the SIS-model, the term “susceptible” merely means that the individual is not infected, so that it can become infected. It does not indicate any degree of susceptibility or resistance of the individual. Individuals ‘move’ between the two states: susceptible individuals can get infected following contact with an infected individual, while infected individuals can recover from infection and become susceptible again. The transition of individuals between these two states occurs at certain rates. The number of individuals that get infected per unit of time, and thus change from S to I, is known as the transmission rate. The number of individuals per unit of time that recover from infection, and thus change from I to S, is the recovery rate.

The transmission rate depends on the transmission rate parameter ($\beta )$, on the number of susceptible individuals ($S$; we will use italics $(S$ and $I$) to indicate the number of individuals in the corresponding state, and regular font ($\mathrm{S}$ and $\mathrm{I}$) to indicate the state of an individual), and on the fraction of the contact individuals that are infected ($\frac{I}{N}$, with $N$ representing the total size of the local population) [24]. Thus,

1 $$Rate\left[\mathrm{S}\to \mathrm{I}\right]=\beta S\frac{I}{N}.$$

The transmission rate parameter $\beta$ is the average number of susceptible individuals that become infected per unit of time by one infected individual in an otherwise fully susceptible population (i.e. $S=N$), and reflects the transmissibility of the infection.

The recovery rate depends on the recovery rate parameter ($\alpha$) and on the number of infected individuals ($I$), i.e.:

2 $$Rate\left[\mathrm{I}\to \mathrm{S}\right]=\alpha I.$$

The recovery rate parameter also determines the average duration of the infectious period, which is equal to $\frac{1}{\alpha }$.

A key parameter in epidemiology is the basic reproduction ratio (${R}_{0}$), which is defined as the average number of secondary infections caused by a single typical infected individual in an otherwise fully susceptible population [25]. ${R}_{0}$ has a threshold function, i.e. when it is greater than 1, an infection may persist in the population, and a considerable fraction of individuals may become infected. When ${R}_{0}$ is less than 1, an infection is guaranteed to die out. Thus, to eradicate an infectious disease, it is essential that interventions reduce ${R}_{0}$ to less than 1.

For the SIS-model, a simple expression for ${R}_{0}$ can be derived using the expressions for the transmission and recovery rate (e.g. [23]). Given that $\beta$ represents the transmission rate for one infected individual in a fully susceptible population, and that the single infected individual has an average infectious period of $\frac{1}{\alpha }$, the number of secondary infections caused by this individual is equal to $\beta /\alpha$. Thus, ${R}_{0}$ is equal to the product of the transmission rate parameter and the duration of the infectious period, i.e.:

3 $${R}_{0}= \frac{\beta }{\alpha }.$$

To simplify the mathematics, we assume a constant value of $\alpha =1$, such that ${R}_{0}=\beta$. Note that we can use $\alpha =1$ without loss of generality because one can always choose a time unit such that $\alpha$ is equal to 1 and, therefore, $\beta$ equals ${R}_{0}$. If ${R}_{0}$ is higher than 1, the SIS-model tends to a situation in which the number of newly infected individuals is equal to the number of recovering individuals, the so-called endemic equilibrium. In that situation, the average numbers of infected and susceptible individuals are stable. The fraction of infected individuals at this equilibrium, i.e. ${I}^{*}/N$, is the endemic prevalence of the infectious disease.

At equilibrium, the transmission rate is equal to the recovery rate:

4 $$\beta {S}^{*}\frac{{I}^{*}}{N}=\alpha {I}^{*}\underset{{R}_{0}= \frac{\beta }{\alpha }}{\iff } {R}_{0}{S}^{*}\frac{{I}^{*}}{N}={I}^{*}.$$

Solving for ${I}^{*}/N$, using ${S}^{*}=N-{I}^{*}$ shows that the endemic prevalence (${P}^{*}={I}^{*}/N$) is determined by ${R}_{0}$:

5 $${P}^{*}=1-\frac{1}{{R}_{0}}.$$

Having defined the fundamental epidemiological model and obtained expressions for ${R}_{0}$ and the endemic prevalence, we will expand the model in the next section by including genetic differences in host resistance.

### Heterogeneous SIS-model with host resistance to wild-type infection

In this section, we present a model for genetic variation in host resistance for a population that is exposed to the wild-type pathogen and present the results of this model. While the term “resistance” might suggest that resistant individuals cannot get infected at all, in practice resistance is rarely all-or-none. Hence, in the following, resistant merely means being less likely to get infected.

When properties of an individual or a pathogen affect the transmission of an infectious disease, they essentially alter the values of the underlying parameters, i.e., $\beta$ and/or $\alpha$, and thus ${R}_{0}$, since these parameters fully encompass the characteristics of the infection in the SIS-model (for examples, see also [26–28]). To include an effect of host resistance on transmission, we decrease ${R}_{0}$ by decreasing $\beta$ (note that it does not matter for the results whether a decrease in ${R}_{0}$ originates from a reduction in $\beta$ or an increase in $\alpha$; see “Discussion”). Furthermore, we assume that, once infected, both non-resistant and resistant hosts are equally likely to infect susceptible individuals. This means that there is no difference in the “infectivity per unit of time” between resistant and non-resistant hosts.

**Fig. 2:**
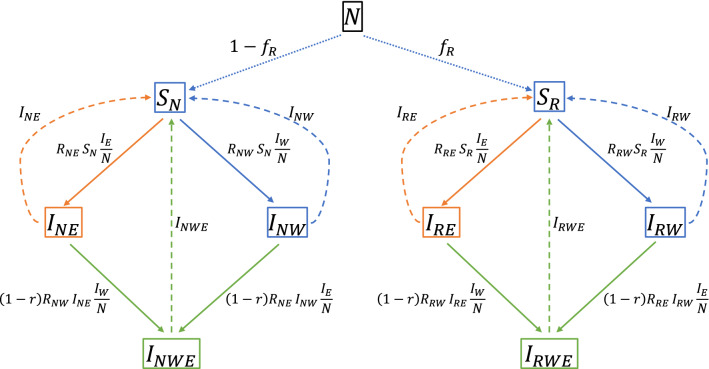
SIS-model with resistant and non-resistant hosts and wild-type and escape-type pathogens. SIS-model with resistant and non-resistant hosts (Blue subsystem), pathogens either of the wild type (subscript W) or that can escape host resistance (subscript E; Orange subsystem), and infection of hosts with both types of pathogen (Green subsystem). For explanation of symbols, see Table 1. ${I}_{W}$ and ${I}_{E}$ denote the total number of individuals infected with a wild-type and escape pathogen, respectively, and are the sum taken over both host types, where ${I}_{W}={I}_{NW}+{I}_{RW}$ and ${I}_{E}={I}_{NE}+{I}_{RE}$ respectively

Now that we have defined how host resistance acts on transmission of the infection, the next step is to define the genetic model for host resistance. Given that derivations for transmission models with many levels of host resistance rapidly become very complex, we will use one of the simplest genetic models for diploid individuals. We assume that resistance is defined by a single locus, which is either fully dominant or fully recessive, such that the host population consists of two types: resistant (with frequency ${f}_{R}$) and non-resistant ($1-{f}_{R}$). Thus, in this model, we have two types of hosts, resistant ($R$) and non-resistant ($N$), and one type of pathogen, the wild-type pathogen ($W$). Using this genetic model, we can set up a SIS-model with two types of hosts, resistant hosts (subscript $R$) and non-resistant hosts (subscript $N$; this is the blue subsystem in Fig. 2 and the full model equations are in Appendix 1). The number of susceptible hosts of each type in the local population are denoted by ${S}_{R}$ and ${S}_{N}$. Analogously, the number of hosts of each type that are infected with the wild-type pathogen (subscript $W$) are denoted by ${I}_{RW}$ and ${I}_{NW}$.

Transmission involves a pair of individuals: an infected donor individual, and a susceptible recipient individual. Here, we assume that the pair-wise transmission rate parameter depends on the resistance genotype of the recipient but not on the genotype of the donor. This is equivalent to the absence of genetic variation in infectivity. Since we use $\alpha =1$, the transmission rate parameters are directly equal to the basic reproduction ratios (Eq. 3). In a contact between an infected and a susceptible (i.e., non-infected) host, these basic reproduction ratios (${R}_{NW}$ and ${R}_{RW}$) are defined for the recipient individual, because we model variation in resistance. The subscript ($N$ or $R$) thus reflects the genotype of the susceptible host ($W$ refers to the wild-type pathogen).

Just like the homogeneous SIS-model, the heterogeneous SIS-model also tends to an equilibrium. However, because of the difference in resistance between individuals, the endemic prevalence in this equilibrium is no longer equal to Eq. (5) because non-resistant individuals are more likely to become infected than resistant individuals. Consequently, at equilibrium, the susceptible individuals are predominantly of the resistant type, which are less likely to become infected. As a result, the overall endemic prevalence at equilibrium is a bit lower than in the model without heterogeneity [26, 27] and is equal to:

6 $${P}_{W}^{*}=\frac{{I}_{NW}^{*}+{I}_{RW}^{*}}{N},$$

and is reached when both host types have reached their equilibrium [4], i.e.:

7 $${R}_{NW}{S}_{N}^{*}\frac{{I}_{W}^{*}}{N}={I}_{NW}^{*}\, \mathrm{and} \,{R}_{RW}{S}_{R}^{*}\frac{{I}_{W}^{*}}{N}={I}_{RW}^{*}.$$

Solving Eqs. (6) and (7) using ${S}_{N}^{*}=\left(1-{f}_{R}\right)N-{I}_{NW}^{*}$ and ${S}_{R}^{*}={f}_{R}N-{I}_{RW}^{*}$, results in equilibrium solutions for the endemic prevalence in both host types and for the overall endemic prevalence in the population. The resulting equations are complex and are given in Appendix 2, together with detailed derivations (Eqs. 20, 21, 22, 23, 24, 25, and 26). We will use figures to illustrate the results.

### Results for the heterogeneous SIS-model with host resistance to wild-type infection

We consider the situation where the wild-type pathogen is endemic in a non-resistant host population, while the infection is absent in a population where all individuals have the resistant genotype due to herd immunity (${R}_{0}<1$). This represents the most beneficial situation for genetic selection for host resistance, because it will lead to eradication of the infection (in this section, escape mutants are ignored). This situation corresponds to ${R}_{NW}>1$, so that ${R}_{0}>1$ when ${f}_{R}=0$, i.e. the infection is endemic in a non-resistant host population, and ${R}_{RW}<1$, so that ${R}_{0}<1$ when ${f}_{R}=1$, i.e. the infection is absent in a resistant host population. Note that ${R}_{RW}<1$ results in the infection to be absent because of herd immunity, but this does not imply that resistant individuals cannot get infected at all (see also results below). We will also show a situation where ${R}_{RW}>1$, to illustrate what will happen when the resistance is not sufficient to fully eradicate the infection.

**Fig. 3:**
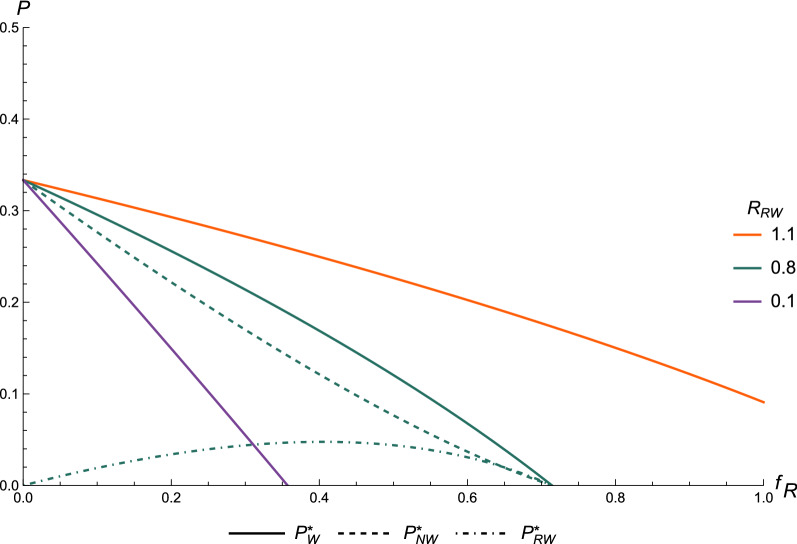
Prevalence of the infection with the wild-type pathogen. Equilibrium prevalence of the infection with the wild-type pathogen (${P}_{W}^{*}=\frac{{I}_{W}^{*}}{N}$), as a function of the frequency of resistant hosts ${f}_{R}$ for three different values of ${R}_{RW}$, ${R}_{NW}=1.5$, such that the endemic prevalence of the wild type in a fully non-resistant population is 0.33 (Eq. 5). For ${R}_{RW}=0.8$, the fractions of infected resistant (${P}_{RW}^{*}=\frac{{I}_{RW}^{*}}{N})$ and non-resistant (${P}_{NW}^{*}=\frac{{I}_{NW}^{*}}{N}$) hosts are also shown

In a population with both resistant and non-resistant individuals, the prevalence and whether the infection is present or not, depend on the frequency of resistant hosts (${f}_{R}$). Figure 3 shows the endemic prevalence of the infection (${P}_{W}^{*}$) in a population that consists of a mix of resistant and non-resistant hosts, as a function of ${f}_{R}$, and for three values of the basic reproduction ratio for resistant hosts (${R}_{RW}$). The basic reproduction ratio for non-resistant hosts (${R}_{NW}$) was set to 1.5. Figure 3 clearly shows that the overall prevalence decreases with increasing frequency of resistant hosts, at a rate that depends on the value of ${R}_{RW}$, i.e. prevalence decreases faster when ${R}_{RW}$ is lower. At a low frequency of resistant hosts, virtually all infected hosts are from the non-resistant type (${P}_{NW}^{*}$; dashed line in Fig. 3), which makes sense given the low frequency of resistant hosts and their lower susceptibility. Note that prevalence is expressed relative to the total population size and not to the number of individuals of a given type, i.e., as ${I}_{RW}^{*}/N$ rather than ${I}_{RW}^{*}/({f}_{R}N)$*.* At a higher frequency of resistant hosts (e.g., ${f}_{R}=0.25$), a larger fraction of the infections occurs in resistant hosts (${P}_{RW}^{*}$; dashed-dotted line in Fig. 3). This happens even when the reproduction ratio of resistant hosts is lower than 1, because the overall ${R}_{0}$ is higher than 1, leading to maintenance of the infection in the population. For ${R}_{RW}$ of 0.1 and 0.8, the overall prevalence decreases to 0 at a certain ${f}_{R}$. This is the frequency of resistant hosts above which the infection is expected to die out, because the greater resistance of the population reduces the overall reproduction ratio of the infection to less than 1 (herd immunity). For ${R}_{RW}$ of 1.1, this point does not exist, because the basic reproduction ratio in a fully resistant population is still above the threshold of 1, implying that the infection will persist in the population.

For ${R}_{RW}<1$, the ${f}_{R}$ at which the infection dies out can be found by realising that the overall ${R}_{0}$ should be higher than 1 for the infection to persist in the population. For our model, the overall basic reproduction ratio is the average of the type-specific reproduction ratios, weighed by the frequencies of both types [28], i.e.:

8 $${R}_{0}=\left(1-{f}_{R}\right){R}_{NW}+{f}_{R}{R}_{RW.}$$

Solving ${R}_{0}=1$ for ${f}_{R}$ results in the upper limit of ${f}_{R}$, below which the infectious disease can persist in the population, i.e.:

9 $${f}_{{R}_{max}}=\frac{{R}_{NW}-1}{{R}_{NW}-{R}_{RW}}.$$

Using the values for ${R}_{NW}$ and ${R}_{RW}$ from Fig. 3, we find solutions for ${f}_{{R}_{max}}$ of $0.5/1.4=0.36$ for ${R}_{RW}=0.1$ and $0.5/0.7=0.71$ for ${R}_{RW}=0.8$. These results represent the minimum frequency of hosts with the resistant genotype in a population that is required to eradicate an infection, which is relevant for selection.

In a closed population, ${{f}_{R}}_{max}$ also sets an upper bound to the invasion possibility of escape mutants, because the infection with the wild-type pathogen dies out when ${f}_{R}>{{f}_{R}}_{max}$. When the wild-type infection has died out, mutants cannot develop anymore in a closed local population, simply because there are no wild-type pathogens to mutate from. The next section provides further details on the important role of ${{f}_{R}}_{max}$ when the model is expanded by allowing for the development of pathogen mutants that can escape host resistance.

### Heterogeneous SIS-model with introduction of an escape mutant

When we want to include pathogen mutants that can escape host resistance into our model, where resistance is not absolute, pathogen escape entails that the mutant pathogen is better able to infect resistant hosts than the wild-type pathogen. We will incorporate one escape mutant into the model, such that there are two pathogen types, a wild-type pathogen ($W$) and an escape mutant ($E$), each of which can infect both host types, albeit the escape mutant will more easily infect a resistant host than the wild-type pathogen. This does not mean that we only consider one type of mutant, it merely means that we will look at the competition between a certain mutant and the wild-type pathogen one at a time. Furthermore, we do not consider the probability that an escape mutation occurs but focus on the case where escape mutants are present, because a mutation will occur sooner or later, as long as the wild-type pathogen is present in the population.

In this section, we assume that infection with one of the two pathogen types offers full cross-resistance against infection with the other type, such that hosts can be infected with only one pathogen type at a time (this assumption will be relaxed in the next section). It means that we consider two additional types of infected individuals, i.e., $N$ and $R$ host types that are infected with the escape ($E$) mutant, such that we have four types of infected individuals in total (the orange subsystem in Fig. 2; note that we still have two types of susceptible individuals). We will model transmissibility of escape mutants by including two additional reproduction ratios in our model, such that transmission between a pair of individuals no longer depends only on the type of susceptible host ($N$ or $R$), but also on the type of pathogen that is carried by the infected host ($W$ or $E$). These two reproduction ratios are for the escape mutant infecting non-resistant or resistant susceptible hosts, denoted by ${R}_{NE}$ and ${R}_{RE}$, respectively. Selection pressure on the pathogen population is then determined by the four reproduction ratios and the frequency of resistant hosts in the population.

Mutants typically arise in a host population that is endemically infected with the wild-type pathogen. Thus, to determine whether an escape mutant can spread in the host population, we need to derive an expression for the reproduction ratio of the escape mutant in a host population in which the wild-type pathogen is at endemic equilibrium. We will call this the invasion reproduction ratio of the escape mutant (${R}_{INV}$). When ${R}_{INV}>1$, the escape mutant can invade a host population that is endemically infected with the wild-type pathogen. When ${R}_{INV}<1$, escape mutants might occur, but they cannot spread.

We can derive ${R}_{INV}$ by applying the definition of ${R}_{0}$, but this time for a population where the wild-type pathogen is endemic, instead of for a fully susceptible population. To find ${R}_{INV}$, given full cross-resistance (i.e. hosts that are infected with one of the pathogen types cannot simultaneously get infected with the other type), we need to multiply the basic reproduction ratios of the escape mutant with the fraction of susceptible individuals (i.e., all individuals of a given genotype that are not yet infected with the wild-type pathogen). These fractions are given by $(1-{f}_{R})-{P}_{NW}^{*}$ for non-resistant hosts, and by ${f}_{R}-{P}_{RW}^{*}$ for resistant hosts. Then, the reproduction ratio for invasion of the escape mutant into a host population that is endemically infected with the wild-type pathogen becomes:

10 $${R}_{INV}={R}_{NE}\left[\left(1-{f}_{R}\right)-{P}_{NW}^{*}\right]+{R}_{RE}\left({f}_{R}-{P}_{RW}^{*}\right).$$

${R}_{INV}$ thus depends on ${f}_{R}$ and the four reproduction ratios, two of which are shown explicitly in Eq. (10) (${R}_{NE}, {R}_{RE}$), while the other two (${R}_{NW}$ and ${R}_{RW}$) are implicit in the expressions for the endemic equilibrium prevalences ${P}_{NW}^{*}$ and ${P}_{RW}^{*}$ (see Eqs. 24 and 25 in Appendix 2).

### Results for the heterogeneous SIS-model with introduction of an escape mutant

**Fig. 4:**
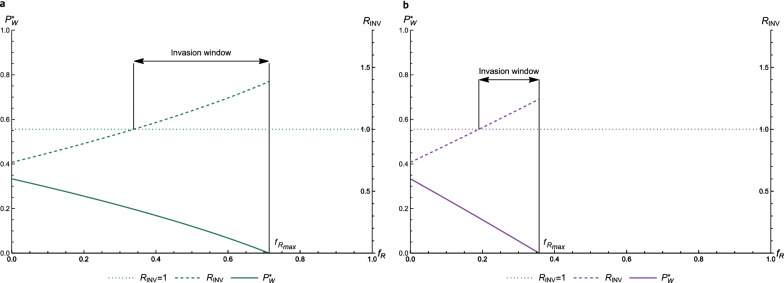
Invasion window with full cross resistance. Reproduction ratio for invasion of an escape mutant in a population endemically infected with the wild-type pathogen as a function of ${f}_{R}$ (${R}_{INV},$ dashed line, right y-axis), for ${R}_{RW}=0.8$ (panel **a**) and ${R}_{RW}=0.1$ (panel **b**). The dotted line shows the threshold value for ${R}_{INV}$ of 1. Hosts can be infected with only one type of pathogen at a time (full cross resistance). Prevalence of the wild-type pathogen as a function of ${f}_{R}$ is given by the solid line, indicated on the left y-axis, comparable to Fig. 3. The window in which there is a risk of invasion of the escape mutant is indicated. ${R}_{NW}=1.5, {R}_{NE}=1.1, {R}_{RE}=1.5$

Figure 4 shows ${R}_{INV}$ (dashed line, right y-axis) as a function of ${f}_{R}$ for reproduction ratios ${R}_{NW}=1.5$, ${R}_{NE}=1.1,{R}_{RE}=1.5$, and ${R}_{RW}=0.8$ (Fig. 4a) or $0.1$ (Fig. 4b). The endemic prevalence of the wild-type pathogen (${P}_{W}^{*}$; solid line; left y-axis) is shown in both panels as a function of ${f}_{R}$, similar to Fig. 3. We chose to simulate ${R}_{RW}<1$, such that the wild-type pathogen will die out in a fully resistant population (see previous section). This is visible in Fig. 4 as the solid line that decreases to ${P}_{W}^{*}=0$. By assumption, the escape mutation comes with a cost in fitness for the pathogen, such that the escape mutant will be outcompeted by the wild-type pathogen in a non-resistant population. However, due to its adaptation to the resistant host, it can persist in a fully resistant population, in contrast to the wild-type pathogen. This is visible in Fig. 4 as the increasing dashed line.

At a certain ${f}_{R}$, the reproduction ratio for invasion of the escape mutant (${R}_{INV}$; dashed line in Fig. 4) crosses the threshold ${R}_{INV}=1$ (indicated by the dotted line in Fig. 4), above which the escape mutant can invade the population. This is a critical point that represents the lower bound of the invasion window, there is a risk of escape mutants invading the (partly) resistant host population when the frequency of resistant hosts is greater than this lower bound. Together with the upper bound of the wild-type pathogen dying out, as defined in the previous paragraph, the point ${R}_{INV}=1$ determines the critical range of the frequency of resistant hosts in which an escape mutant pathogen can both arise (${P}_{W}^{*}>0$) and invade (${R}_{INV}>1$). This range of ${f}_{R}$ is indicated as the ‘invasion window’ in Fig. 4.

Comparing panels (a) and (b) of Fig. 4, we see that a stronger effect of the resistance gene of the host (lower ${R}_{RW}$) not only results in a lower ${f}_{R}$ that is required to eradicate the wild-type pathogen ($0.36<0.71$), but also in a narrower invasion window, because of a lower upper bound. A stronger effect of the resistance gene (i.e., lower ${R}_{RW}$) thus reduces the risk of invasion of escape mutants because it results in a smaller invasion window. A smaller invasion window can be passed more quickly by artificial selection of the host population.

In summary, in this section we have derived an expression for the reproduction ratio for the invasion of an escape mutant in hosts that are endemically infected with the wild-type pathogen, assuming that hosts can be infected with only one pathogen at a time (full cross-resistance). In the next section, we will relax this assumption and expand our model to allow for infection of hosts with both types of pathogen at the same time.

### Heterogeneous SIS-model, allowing for double infections

Although the two pathogen types are closely related, infection with the one type may not provide full resistance to infection with the other type. Coexistence of different strains (incomplete cross-resistance) is common for many bacteria (e.g. [29–31]). However, for viruses such as influenza A, infection with the wild-type pathogen may give substantial resistance to infection with the escape mutant and vice versa [32]. If cross-resistance is not complete, hosts that are already infected with one pathogen type can get infected with another as well. Thus, the possibility of double infection leads to an additional transmission route, from singly-infected to doubly-infected hosts. If some cross-resistance is present, this leads to a lower rate for a second infection compared to the rate of first infection. We will model this effect by including a cross-resistance factor ($1-r$) in the transmission rate from singly- to doubly-infected hosts (the green subsystem in Fig. 2). Parameter $r$ takes values between 0 and 1; 0 for no cross-resistance and 1 for full cross-resistance (no double infections occur). For example, non-resistant hosts that are infected with the wild-type pathogen get infected with the escape mutant at rate $(1-r){R}_{NE}{I}_{NW}\frac{{I}_{E }}{N}$. Once infected, we assume that doubly-infected hosts are equally likely to infect a susceptible host with one of the two pathogen types as singly-infected hosts.

To determine whether an escape mutant can invade under these conditions, we need to adapt Eq. (10) because susceptible individuals as well as individuals that are already infected with the wild-type pathogen can become infected by the escape mutant. The equation for the invasion reproduction ratio of an escape mutant in a population that is endemically infected with the wild-type pathogen then becomes:

11 $${R}_{INV}\,=\,{R}_{NE}\left[(1-{f}_{R})-{P}_{NW}^{*}+(1-r){P}_{NW}^{*}\right]+{R}_{RE}\left[{f}_{R}-{P}_{RW}^{*}+(1-r){P}_{RW}^{*}\right].$$

This expression allows us to investigate how invasion of an escape mutant is affected by incomplete cross-resistance.

### Results for the heterogeneous SIS-model that allows for double infections

**Fig. 5:**
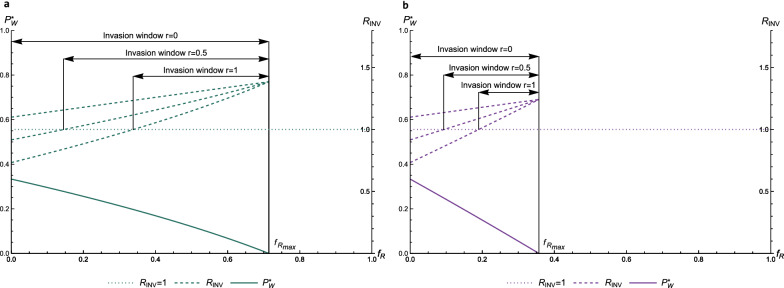
Invasion window for different levels of cross-resistance. Reproduction ratio for invasion of an escape mutant in a population endemically infected with the wild-type pathogen as a function of ${f}_{R}$ (${R}_{INV},$ dashed lines, right y-axis), for cross resistance (r) levels of 0, 0.5 and 1 and for ${R}_{RW}=0.8$ (panel **a**) and ${R}_{RW}=0.1$ (panel **b**). The dotted line shows the threshold value for ${R}_{INV}$ of 1. Prevalence of the wild-type pathogen as a function of ${f}_{R}$ is given by the solid line, indicated on the left y-axis. The invasion window for the different cross-resistance levels is indicated. ${R}_{NW}=1.5, {R}_{NE}=1.1, {R}_{RE}=1.5$

Figure 5 shows the effect of incomplete cross-resistance on the invasion window of the escape mutant. Figure 5 is similar to Fig. 4 (which implicitly has $r=1$), but now ${R}_{INV}$ is shown for three levels of cross-resistance (0, 0.5 and 1). Reproduction ratios are the same as those used in Fig. 4. Incomplete cross-resistance ($r<1$) increases the width of the invasion window. When $r=0$, the window covers the whole range in ${f}_{R}$ where the wild-type pathogen is present. This happens because ${R}_{NE}$ is greater than 1 and, when $r=0$, all hosts infected with the wild-type pathogen can still get infected with the escape mutant as well, because, in this case, there is no competition between the pathogen types. As without double-infected hosts, a lower ${R}_{RW}$ (Fig. 5b) decreases the width of the invasion window. In the next section, we will further investigate how the reproduction ratios affect the width of the invasion window at different levels of cross-resistance.

### Factors affecting the width of the invasion window

As shown in the previous paragraphs, the invasion opportunity of escape mutants in a host population that is genetically selected for resistance is restricted by two bounds: (1) a lower bound that represents the degree of resistance of the population at which the invasion reproduction ratio (${R}_{INV}$) becomes greater than 1, and (2) an upper bound that represents the point above which the wild-type pathogen dies out (${{f}_{R}}_{max}$, Eq. 9). Since we obtained an expression for ${{f}_{R}}_{max}$, we tried to solve ${R}_{INV}=1$ for ${f}_{R}$ algebraically, to determine the effect of the four reproduction ratios and the level of cross-resistance on the width of the invasion window. Unfortunately, this resulted in a closed form solution only when there is either no cross-resistance or full cross-resistance ($r=0$ or $r=1$). Thus, we will investigate the width of the invasion window numerically by taking one set of values for the four reproduction ratios as the default scenario (${R}_{NW}=1.5, {R}_{RW}=0.8, {R}_{NE}=1.1, {R}_{RE}=1.5$), and then vary one of them at a time (except ${R}_{NW}$). For cross-resistance, $r$ will take values of 0, 0.5, and 1.

**Fig. 6:**
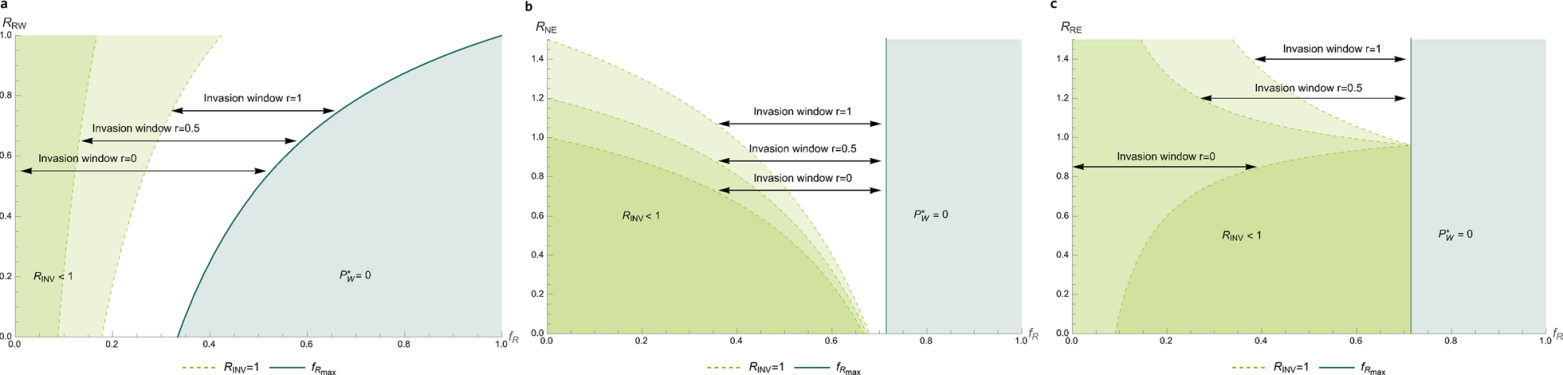
Size of the invasion window for different values of ${R}_{RW}$. Value of ${f}_{R}$ for the lower bound of the invasion window, ${R}_{INV}=1$, as a function of the basic reproduction ratio of the wild-type pathogen in resistant hosts (${R}_{RW}$) (panel **a**), the basic reproduction ratio of the escape mutant in non-resistant hosts (${R}_{NE}$) (panel **b**), and the basic reproduction ratio of the escape mutant in resistant hosts (${R}_{RE}$). For levels of cross resistance of 0, 0.5 and 1 (dashed, light green lines). In panel (**a**), for cross resistance of 0, the line lies on the y-axis. The value of ${f}_{R}$ from which the wild-type pathogen dies out (upper bound of the invasion window) is shown by the solid green line. The invasion window for the different cross-resistance levels is indicated by the arrows. For ${R}_{NW}=1.5$, ${R}_{RW}=0.8$ (panels **b** and **c**), ${R}_{NE}=1.1$ (panels **a** and **c**), and ${R}_{RE}=1.5$ (panels **a** and **b**)

Figure 6a shows the size of the invasion window as a function of the frequency of resistant hosts and the basic reproduction ratio of the wild-type pathogen in resistant hosts (${R}_{RW}$). We can see that the window becomes smaller with decreasing ${R}_{RW}$, as was already visible in Figs. 4 and 5. As stated before, this implies that a stronger effect of the resistance gene (i.e., lower ${R}_{RW}$) decreases the risk of invasion of an escape mutant, because the wild-type pathogen goes extinct sooner. The decreasing width of the window with a lower ${R}_{RW}$ is mainly caused by the effect on ${{f}_{R}}_{max}$ (solid dark green line in Fig. 6a), which becomes closer to 1 with increasing ${R}_{RW}$. Hence, a change in ${R}_{RW}$ mainly impacts the upper bound of the invasion window.

The lower bound is less affected by a change in ${R}_{RW}$; and especially for low $r$, the line ${R}_{INV}=1$ is almost vertical in Fig. 6a. This happens because at lower levels of cross-resistance, there is less competition between the two pathogen types, since (part of) the hosts that are infected with one pathogen type can also become infected with the other type. In the most extreme case, when there is no cross-resistance ($r=0$), the lower bound is not affected by the frequency of resistant hosts (Fig. 6a). This means that ${R}_{RW}$, and thus the endemic equilibrium prevalence of the wild-type pathogen, has no effect on the risk of invasion of an escape mutant when $r=0$.

We restricted the y-axis of Fig. 6a to a maximum of 1, because the pathogen can persist in resistant hosts when ${R}_{RW}>1$, and it is thus impossible to eradicate the wild-type pathogen. Genetic selection for infectious disease resistance will in that case not be sustainably beneficial because escape mutants will eventually occur, even at a high frequency of resistant hosts. Thus, to prevent invasion of escape mutants, ${R}_{RW}$ needs to be less than 1, either through the effect size of the resistance genes or by taking additional measures in combination with genetic selection to achieve ${R}_{RW}$ less than 1.

Figure 6b shows the size of the invasion window as a function of the frequency of resistant hosts and the basic reproduction ratio of the escape mutant in non-resistant hosts (${R}_{NE}$). The value of ${R}_{NE}$ relative to ${R}_{NW}$ reflects the fitness costs for the escape mutant in a non-resistant host population, relative to the wild-type pathogen. If ${R}_{NE}$ is much lower than ${R}_{NW}$, the escape mutant spreads much less in non-resistant hosts than the wild-type pathogen, i.e., the escape mutation comes with high fitness costs (low *y*-axis value). If ${R}_{NE}$ is close or equal to ${R}_{NW}$, differential fitness costs are low or absent and the spread of the escape mutant in non-resistant hosts is similar to the spread of the wild-type pathogen (high *y*-axis value). Consequently, the size of the invasion window increases as ${R}_{NE}$ moves closer to ${R}_{NW}$. At a certain ${R}_{NE}$, the invasion window covers the whole range of ${f}_{R}$ in which the wild-type pathogen can persist. When there is full cross-resistance (top dashed line, $r=1$), this point occurs when ${R}_{NE}$ is equal to ${R}_{NW}$ (= 1.5), meaning that the escape mutant spreads equally well as the wild-type pathogen in non-resistant hosts. With no cross-resistance (bottom dashed line, $r=0$), this point occurs when ${R}_{NE}$ is equal to 1. If there is no cross-resistance, the presence or absence of the wild-type pathogen has no effect on the spread of the escape mutant and, thus, a basic reproduction ratio greater than 1 is sufficient for the mutant to persist. We can also see in Fig. 6b that the invasion window stays relatively small for lower values of ${R}_{NE}$ and only covers a range in ${f}_{R}$ of about 0.05 when ${R}_{NE}$ is close to 0. This indicates that it is extremely difficult for escape mutants to invade when they experience high fitness costs in non-resistant hosts.

Figure 6c shows the size of the invasion window as a function of the frequency of resistant hosts and of the basic reproduction ratio of the escape mutant in resistant hosts (${R}_{RE}$). The size of the invasion window increases with ${R}_{RE}$, but less so when cross-resistance is high. If there is a little cross-resistance, the mutant can already invade if ${R}_{RE}$ is slightly less than 1 (top dashed lines for $r=0.5$ and $r=1$ in Fig. 6c), because ${R}_{NE}>1$ (1.1) and ${R}_{RW}<{R}_{RE}$ in that case, i.e., host resistance is more effective against the wild-type pathogen than against the escape mutant. Thus, in a very small range of ${f}_{R}$, “escape” mutants might invade even though their reproduction ratio in resistant hosts is less than 1. The increasing slope of the line ${R}_{INV}=1$ when there is no cross-resistance (bottom dashed line in Fig. 6c, $r=0$), mainly results from ${R}_{NE}$ being greater than 1. Figure 6b shows that at an ${R}_{NE}$ of 1.1 and no cross-resistance, the invasion window covers the full range in ${f}_{R}$ from 0 to ${{f}_{R}}_{max}$. So, in a population that consists of mainly non-resistant hosts (left side of Fig. 6), the escape mutant will be well able to invade, as explained in a previous paragraph. At a higher frequency of resistant hosts, as with the wild-type pathogen, the transmission of the escape mutant in resistant hosts becomes more and more determining for the invasion probability, indicated by ${R}_{RE}$. If ${R}_{RE}$ is less than 1, host resistance is not only effective against the wild-type pathogen but also against the “escape” mutant, which is evident for the increase in the lower bound of the invasion window (the line ${R}_{INV}=1$) with increasing ${f}_{R}$ for $r=0$ in Fig. 6c. The combination of reproduction ratios of the escape mutant in non-resistant and resistant hosts, together with cross-resistance, thus determines its opportunity to invade.

## Discussion

Using a mathematical model of disease transmission, we showed that an infectious disease can be eradicated by increasing the frequency of genetically-resistant hosts to a sufficient level, if the reproduction ratio of the wild-type pathogen in resistant hosts is less than 1. Eradication was achieved by herd immunity in the local population, when ${R}_{0}$ falls below a value of 1, which requires neither that all individuals in the local population are resistant, nor that resistant individuals are fully resistant. The minimum frequency of resistant hosts needed to eradicate an infection (${{f}_{R}}_{max}$, Eq. 9) is a function of the reproduction ratios of the pathogen in resistant and non-resistant hosts.

As long as the wild-type pathogen is not eradicated (i.e., the frequency of resistant hosts is not high enough), escape mutants of the pathogen can arise and invade the population. To determine whether escape mutants can invade or not, we derived a reproduction ratio for escape mutants in a population that is endemically infected with the wild-type pathogen ($${R}_{INV}$$, Eq. 11). Invasion of escape mutants then depends on the reproduction ratios of the wild-type pathogen in resistant and non-resistant host types (through the endemic prevalence), the frequency of resistant hosts, and the basic reproduction ratios of the escape mutant in non-resistant and resistant hosts. We identified an ‘invasion window’, i.e. the range for the frequency of resistant hosts within which there is a risk of escape mutant invasion. The lower bound of this window is the frequency of resistant hosts above which the reproduction ratio of the invading escape mutant ($${R}_{INV})$$ becomes greater than 1. The upper bound of the invasion window is the frequency of resistant hosts above which the wild-type pathogen dies out ($${f}_{{R}_{max}}$$). The invasion window is wider, implying more opportunity for escape mutants to invade, when the resistance allele has a smaller effect and when hosts can get infected by both types of pathogen at the same time (i.e., cross-resistance is not complete).

### Model assumptions

In our model, we assumed that host resistance was determined by a single, fully dominant or fully recessive locus. This assumption resulted in a relatively simple model, with only two types of hosts, which helped in the interpretation of the results. Although the assumption of a single locus determining resistance might only be realistic for very few cases, our predictions will also hold if a quantitative trait locus (QTL) explains a considerable part of the genetic variation, preferably strong enough to bring ${R}_{RW}$ below 1. Such a major QTL was for instance found for infectious pancreatic necrosis (IPN) resistance in Atlantic salmon [33]. In most other cases, infectious disease resistance is likely a polygenic trait that is affected by many QTL, each explaining only a small part of the genetic variation, as found for instance by genome-wide association studies (GWAS) for mastitis and digital dermatitis resistance in dairy cattle [34, 35]. With polygenic resistance, eradication of the wild-type pathogen will also be needed to prevent invasion of escape mutants. Hence, our results are relevant for that case also. However, if polygenic resistance comes with multiple resistance mechanisms, pathogens will be less likely to evolve escape mutations to all mechanisms, and predictions become more complex. We will further elaborate on this in the “Implications for animal breeding” section.

Key to the general patterns that emerge from our models are the values for the reproduction ratios. We assumed ${R}_{NW}>1$, such that the wild-type pathogen is endemic in non-resistant hosts, and ${R}_{RW}<1,$ such that resistance is effective against the wild-type pathogen. Thus, before starting selection for increased host resistance, it is important to know the values of ${R}_{NW}$ and ${R}_{RW}$. Values for ${R}_{NW}$ for a specific infection are probably already available or can be estimated from field data, e.g. based on the endemic prevalence of the infection (Eq. 5). Values for ${R}_{RW}$ could be obtained from transmission experiments, such as those that are used to estimate the effectivity of vaccines [36–39], or from knowledge of QTL-effects on resistance (susceptibility) or recovery, for example from GWAS.

Estimating the basic reproduction ratios for the escape mutant is more difficult, since these mutants have not yet emerged when selection starts. Nevertheless, in our opinion, some general predictions of the typical properties of escape mutations can be made. We argue that the wild-type pathogen has likely evolved over a long time to be adapted to the non-resistant host. This implies that a typical escape mutant will probably be less well adapted to non-resistant hosts, and thus will have a lower reproduction ratio than the wild-type pathogen in these hosts (${R}_{NE}<{R}_{NW}$). In other words, the escape mutation typically comes with a cost in pathogen fitness in non-resistant hosts, which is expected and often observed for such mutations [40, 41]. In resistant hosts, the escape mutant will, by definition, have a higher reproduction ratio than the wild-type pathogen (${R}_{RE}>{R}_{RW}$). However, since the two pathogens are closely related and the wild-type pathogen is adapted to the non-resistant host, it is probably unlikely that ${R}_{RE}$ will exceed the reproduction ratio of the wild-type pathogen in non-resistant hosts (${R}_{RE}\le {R}_{NW}$).

We modelled pathogen characteristics via the transmission rate parameter ($\beta$), assuming a recovery rate ($\alpha$) of 1, which allowed us to present the results in terms of reproduction ratios. Since most livestock diseases of interest to animal breeding have a low mortality, we assumed that the pathogen does not kill the host. Thus, the infectious period does not end with death of the host, but with its recovery. A brief investigation showed that the reproduction ratios fully capture variation in both the transmission and recovery rate parameters in our SIS-model (results not shown). Thus, for the possibility of a mutant to invade, it does not matter whether a lower reproduction ratio in resistant hosts is caused by reduced transmissibility of the pathogen (lower $\beta$) compared to in non-resistant hosts, by a greater recovery rate of the host (higher $\alpha$), or by a combination of both. This result implies that the assumption of $\alpha =1$ does not restrict the generality of our results, but merely served to simplify the mathematics. Furthermore, our results are not restricted to situations in which a trade-off between recovery and transmissibility exists; given the reproduction ratios used in our model, results do not depend on the nature of such a trade-off, although evolution of pathogen virulence may depend on this trade-off (see below). With incomplete cross-resistance, the generality of our results with respect to variation in the transmission and recovery rate parameters holds if the recovery rate parameter of hosts infected with both pathogen types is identical to the recovery rate parameter of hosts that are infected with the escape mutant only. Further research is needed to assess whether this condition is realistic.

Another important assumption underlying our results is that there is no continuous import of infectious material or infectious animals from outside the herd. If infectious material frequently enters from outside, minor outbreaks of the wild-type pathogen might still occur in a herd that is genetically-selected for higher resistance. Here, a minor outbreak refers to the introduction of an infection in a population leading to only a few infected individuals, after which the infection dies out [42]. Escape mutants may then also emerge and invade from these minor outbreaks. The assumption of a closed or semi-closed local population holds or can hold for many livestock diseases, depending on farm management. Even if pathogens are transmitted through the environment, the population might still be closed, as long as the infectious material is excreted in the environment by animals from the same farm. This is for instance most likely the case for some important pathogens that cause mastitis and claw infection digital dermatitis [43, 44], but not for bovine tuberculosis, where badgers or other wildlife species are an important environmental infection source [45].

### Pathogen evolution and eradication

We deliberately ignored the evolutionary dynamics in the pathogen population, which have been studied extensively (e.g. [28, 46–52]). In these studies, interest is usually in host mortality, or virulence, of the pathogen at its evolutionary optimum, and results show that whether or not a mutant strain will emerge and invade strongly depends on the model assumptions. Our interest, however, was to identify the range of host population resistance within which an escape mutant can invade. This different interest mainly follows from the human control on genetic selection, animal movement, and isolation or removal of infectious individuals in livestock populations, as opposed to human or natural populations. Moreover, the more closed and localised structure of livestock populations makes eradication of an infection, especially locally, much more feasible in livestock than in human populations, which is illustrated by the disease-free status of many countries and regions for animal infections such as the Pseudorabies virus (Aujeszky’s disease) and bovine tuberculosis [13]. Our recent results show that such eradication by genetic selection is much more promising than commonly believed [4, 6]. Hence, similar to the use of vaccination, genetic selection in livestock can, at least in principle, be used to eradicate a pathogen from the (local) population. When the objective is eradication, interest is not in the evolution of pathogens to an optimum, but in avoiding the invasion of escape mutants before (local) eradication has been achieved.

Our model with two types of hosts is essentially comparable to models that are used to investigate the evolutionary consequences of vaccination on the pathogen. Such models have received much more attention in the literature than models of the evolutionary consequences of artificial genetic selection of the host population. Gandon and Day [53] mention two main approaches that are taken in such evolutionary vaccination studies: (1) the modelling of the invasion of escape strains of the pathogen, similar to our approach, and (2) the modelling of evolutionary changes in the virulence of the pathogen. We will discuss some examples of both approaches below and point at important similarities and differences with our study.

Concerning the first approach, Gandon and Day [53] show expressions for the critical vaccination coverage that is required to eradicate an infection, analogous to our ${f}_{{R}_{max}}$ (Eq. 9), and for the reproduction ratio of invasion of an escape mutant, analogous to our ${R}_{INV}$ (Eq. 11). Manet al. [54] conclude that replacement of the wild type human papillomavirus by a mutant strain depends on the trade-off between the amount of cross-immunity (our parameter $r$) and cross-protection of the vaccine against the mutant strain (i.e. the ratio $\frac{{R}_{RE}}{{R}_{RW}}$ in our model), which closely corresponds to our findings.

As discussed above, an important difference between our study and many other studies on pathogen escape is that we focus on eradication of the infection. In the model of McLean [55], who investigated why escape mutants did not arise in several vaccination campaigns, the vaccine-favoured strain (i.e. the escape mutant) takes over sooner or later, such that the infection is never eradicated. Similarly, Magori and Park [56] investigated how different types of imperfect vaccines influence the rate of spread of mutant strains with a fixed vaccination coverage of 0.25. In their model, the infection was not eradicated, because the reproduction ratio of the wild-type pathogen was still above 1 at their assumed vaccination coverage. The main reason why these vaccination studies have not considered eradication is probably because they focus on human infections, where (local) eradication is much less feasible than in livestock (see above).

The second approach that has been used to model evolutionary changes in pathogen virulence, differs more from the approach used in our study. The main interest in that approach is in pathogens that may evolve to become more harmful after vaccination of the host population. A prominent example from livestock of such a change is the evolution of Marek’s disease virus, a highly contagious virus of poultry. Vaccination of poultry flocks with a vaccine that did not stop transmission of the virus resulted in a new variant of the virus that was more harmful to both vaccinated and unvaccinated host [16]. An example from the human population is the evolution of the bacterium that causes whooping cough (pertussis) following vaccination with a vaccine that reduces disease symptoms but does not prevent transmission of the bacterium [57]. In both these cases, the pathogen became more harmful after the vaccination campaign, which relates back to the virulence-transmission trade-off, which is extensively discussed in the evolutionary literature (see [58] for a review). When vaccination reduces clinical symptoms but does not stop transmission, more transmissible strains of the pathogen can evolve, without paying the cost of killing the host rapidly. However, when the aim of the disease control strategy is to eradicate infection, as is the focus in our study, difficulties associated with increased virulence of the pathogen are prevented. Nevertheless, the above examples stress the importance of further research into the sustainability of selection for tolerance or resilience [59–62], where the goal is to reduce the impact of disease on production and not necessarily to stop transmission.

## Implications for animal breeding

Our results show that a window of invasion for escape mutants can be identified for the frequency of resistant hosts in a local population. Typically, this invasion window does not cover the entire range of the frequency of resistant hosts. Thus, an implication of the results (e.g. Fig. 4) is that, in a closed population, the frequency of animals with the resistance gene should be increased as rapidly as possible through the window, i.e., from the lower bound where ${R}_{INV}>1$ to the upper bound of eradicating the wild-type pathogen (${{f}_{R}}_{max}$). This allows the infection to be eradicated while minimizing the risk of invasion of escape mutants that nullify the effects of selection. Note that an increase of resistant hosts can also lead to a considerable decrease in prevalence, even when the infection is not totally eradicated. Although this seems desirable at first sight, because the number of individuals affected by the infection is largely reduced, it introduces the risk of invasion of escape mutants and this risk remains in the long term.

Here, a rapid increase in the frequency of resistant hosts refers to a rate of increase that is faster than the rate at which escape mutants can develop in the pathogen population. Consequently, current rates of genetic improvement of host resistance may be fast enough when the target infection concerns a slowly evolving macroparasite. For example, selection for a single resistance gene of sheep against gastro-intestinal nematode infection, might not cause adaptation of the nematodes for up to 20 years [63]. However, for rapidly evolving microparasites, the current speed of genetic improvement of host resistance will likely not be fast enough. The strategies that we discuss in the following section to bridge the invasion window may therefore be most relevant to microparasitic infections.

One can think of different strategies to bridge the invasion window. Local livestock populations that are relatively small and largely isolated from other such populations are most suitable for such strategies, because of the possibility to control animal movement and contacts between farms. A possibility to achieve a high frequency of resistant hosts in a local population could be to base herd composition on the resistance of the individual animals, such that herds are either resistant enough to eradicate the infection (i.e., the frequency of the resistant hosts is greater than ${{f}_{R}}_{max}$) or that the frequency of resistant hosts stays below the point above which the escape mutant can invade (i.e. ${R}_{INV}$ stays less than1). This is probably most feasible in cases where the breeding population is separated from the production population, and where all animals in production herds are replaced at once, such as in poultry and sometimes in fattening pigs, as opposed to dairy cattle for example. Although the increase in resistance will necessarily be more gradual in the nucleus selection lines of the former species, the risk of escape mutants emerging seems limited since the selection lines are usually free from clinically important infectious diseases (specific pathogen-free).

When replacement occurs gradually, as in cattle, preventing escape mutants from invading will be much more challenging. If feasible, one could consider separating resistant from non-resistant animals, as in the previous strategy, such that, in fact, two herds are created; one in which the infection with the wild-type pathogen is still endemic but escape mutants cannot invade because the level of resistance is too low, and one in which the wild-type pathogen is eradicated because of the high level of resistance.

Another option would be to increase (local) population resistance rapidly by putting high selection pressure on the resistance trait or by sorting the genetic material, e.g., by using only the most resistant sires on a certain farm. A complicating factor when breeding an endemically infected population for eradication of the pathogen, is that the infection can still be present for some time after the population reaches the point when eradication is expected, before it dies out [64]. During that period, a risk of invasion of escape mutants continues because there is substantial selection pressure on the pathogen population to escape the high frequency of resistant hosts. Finding and removing or isolating infected individuals could then be a way to accelerate eradication. A similar approach is taken when eradicating infections by vaccination (e.g. [14, 65–67]). Note that the ongoing infection as discussed above is not visible in our deterministic model. Similarly, but with a more favourable outcome, the infection might, by chance, die out before the point where eradication is expected.

To reach eradication as soon as possible, it is key that ${R}_{RW}$ is as low as possible (see Fig. 3). Although it might be difficult to influence the size of the effect of resistance genes, it is important that the effect is at least large enough to decrease ${R}_{RW}$ below 1. Thus, when for example multiple QTL for host resistance are identified, selection should preferably increase their frequency simultaneously, instead of one by one. It might also help to include multiple traits in the breeding goal that are focused on reducing infection prevalence, i.e. infectivity and recovery, in addition to only resistance, as we modelled here. Moreover, other interventions against the infection can assist in decreasing ${R}_{RW}$ rapidly to a value less than 1, such as removal of infected animals, vaccination, or general hygienic measures. Thus, a combination of genetic selection for infectious disease resistance and other interventions will be most effective, both in eradicating the wild-type pathogen and in preventing escape mutant invasion.

All strategies described above are also relevant to gene-edited resistance, including the use of other interventions to assist in decreasing the reproduction ratio to a value below 1 [68]. Resistance to the porcine reproductive and respiratory syndrome virus is a clear example where gene-editing might be an interesting strategy for infection control [68], although it is questionable whether many more of such cases exist.

As discussed by Bishop and MacKenzie [64], it is probably more difficult for pathogens to find escape strategies for polygenic disease resistance traits than for monogenic traits, especially when escape from polygenic resistance requires multiple mutations in the pathogen. This expectation is supported by the results of a selection experiment of Kemper et al. [69] in which nematodes showed no adaptation to long-term exposure to polygenic resistant sheep. This phenomenon is more widely observed and exploited in plant breeding, where sustainable strategies for disease resistance breeding consist of combining multiple resistance genes in a cultivar, preferably with different mechanisms of action [70]. Combining multiple genes with different mechanisms of resistance may again be most feasible for species with a separate breeding population. It requires that the breeding population is not exposed to the pathogen before all individuals have at least two genes with different mechanisms of resistance, otherwise pathogens would still be able to evolve resistance to each mechanism one-by-one. However, our quantitative predictions are not easily translated to host resistance based on multiple mechanisms, since this would require a considerable expansion of the model to account for the different mechanisms of resistance and pathogen escape strategies.

In livestock genetic improvement, selection for an index consisting of estimated breeding values for many traits has become the standard [71]. For ordinary quantitative genetic traits, such multi-trait index selection [72] is superior to selection for several traits, one trait at a time, for example over generations (tandem selection), or within generation using independent selection thresholds for each trait (independent culling levels) [73]. Multi-trait selection typically creates small steps of improvement for each trait. However, when the breeding goal includes resistance to an infectious disease, gradual improvement by multi-trait selection will cause the host population to remain in the invasion window for a long time, such that emerging escape mutants might have ample opportunity to invade. Thus, our results give at least some reason to think that multi-trait selection might be suboptimal when infectious disease resistance is part of the breeding goal. The best documented and undisputed case where long-term weak selection against pathogens has resulted in the evolution of escape mutants, is the long-term use of low-doses of antibiotics. That practice has strongly supported the evolution of antibiotic-resistant bacteria, which poses a threat to the availability of effective antibiotics in the long term [74–76]. In a sustainable strategy, resistance is strong enough to bring the reproduction ratio of the wild-type pathogen below 1 (${R}_{RW}<1$). This resembles the use of high doses of antibiotics, which is known to be effective in preventing the evolution of antibiotic resistant strains [75]. For animal breeding, this would correspond to applying the total genetic selection differential for infectious disease resistance within a single or a few generations, at least locally, instead of accumulating it gradually over many generations of multi-trait selection. Although the similarity to the use of low doses of antibiotics clearly questions the sustainability of multi-trait selection when resistance is part of the breeding goal, further (experimental) research is definitely needed here.

## Conclusions

Here, we showed that genetic selection for infectious disease resistance determined by a single locus in a closed livestock population typically provides an opportunity for escape mutants of pathogens to invade the host population. Given the reproduction ratios of the infectious pathogens, we identified the range for the frequency of resistant hosts in the population within which there is this risk of invasion. As long as there is no import of infectious material from outside the closed population, this ‘invasion window’ extends from the frequency of resistant hosts at which the reproduction ratio of the escape mutant in a host population that is endemically infected with the wild-type pathogen becomes greater than 1, until the frequency of resistant hosts above which the wild-type pathogen is expected to die out. To prevent invasion of escape mutants in closed populations, the frequency of resistant hosts should thus be increased faster through this window than escape mutants can develop in the pathogen population. The current, very gradual, multi-trait selection approach in animal breeding might thereby pose a risk of invasion of escape mutants. A possible strategy to prevent the invasion of escape mutants is to place animals into herds based on their genetic resistance, such that herds stay out of the invasion window on either side. Combining such a sustainable selection strategy with other interventions that reduce transmission of the pathogen will help to prevent invasion of escape mutants and to eradicate an infection.

## Data Availability

No data were used for this study.

## Appendix

### Appendix 1: Model equations

12 $$\frac{d{S}_{N}}{dt}=\left({I}_{NW}+{I}_{NE}+{I}_{NWE}\right)-{R}_{NW}{S}_{N}\frac{{I}_{W}}{N}-{R}_{NE}{S}_{N}\frac{{I}_{E}}{N},$$

13 $$\frac{d{S}_{R}}{dt}=\left({I}_{RW}+{I}_{RE}+{I}_{RWE}\right)-{R}_{RW}{S}_{R}\frac{{I}_{W}}{N}-{R}_{RE}{S}_{R}\frac{{I}_{E}}{N},$$

14 $$\frac{d{I}_{NE}}{dt}\,=\,{R}_{NE}{S}_{N}\frac{{I}_{E}}{N}-\left(1-r\right){R}_{NW}{I}_{NE}\frac{{I}_{W}}{N}-{I}_{NE},$$

15 $$\frac{d{I}_{NW}}{dt}\,=\,{R}_{NW}{S}_{N}\frac{{I}_{W}}{N}-\left(1-r\right){R}_{NE}{I}_{NW}\frac{{I}_{E}}{N}-{I}_{NW},$$

16 $$\frac{d{I}_{NWE}}{dt}\,=\,\left(1-r\right){R}_{NW}{I}_{NE}\frac{{I}_{W}}{N}+\left(1-r\right){R}_{NE}{I}_{NW}\frac{{I}_{E}}{N}-{I}_{NWE},$$

17 $$\frac{d{I}_{RE}}{dt}\,=\,{R}_{RE}{S}_{R}\frac{{I}_{E}}{N}-\left(1-r\right){R}_{RW}{I}_{RE}\frac{{I}_{W}}{N}-{I}_{RE},$$

18 $$\frac{d{I}_{RW}}{dt}\,=\,{R}_{RW}{S}_{R}\frac{{I}_{W}}{N}-\left(1-r\right){R}_{RE}{I}_{RW}\frac{{I}_{E}}{N}-{I}_{RW},$$

19 $$\frac{d{I}_{RWE}}{dt}\,=\,\left(1-r\right){R}_{RW}{I}_{RE}\frac{{I}_{W}}{N}+\left(1-r\right){R}_{RE}{I}_{RW}\frac{{I}_{E}}{N}-{I}_{RWE}.$$

### Appendix 2: Prevalence of wild-type pathogen in resistant and non-resistant hosts

At equilibrium:

 20 $${R}_{NW}{S}_{N}^{*}\frac{{I}_{W}^{*}}{N}={I}_{NW}^{*}\,\mathrm{and}\,{R}_{RW}{S}_{R}^{*}\frac{{I}_{W}^{*}}{N}={I}_{RW}^{*}.$$

Given that:

$${S}_{N}^{*}=\left(1-{f}_{R}\right)N-{I}_{NW}^{*},$$

$${S}_{R}^{*}={f}_{R}N-{I}_{RW}^{*},$$

and

$$\frac{{I}_{W}^{*}}{N}={P}_{W}^{*},$$

 21 $${R}_{NW}{P}_{W}^{*}\left(\left(1-{f}_{R}\right)N-{I}_{NW}^{*}\right)={I}_{NW}^{*} \mathrm{and} {R}_{RW}{P}_{W}^{*}\left({f}_{R}N-{I}_{RW}^{*}\right)={I}_{RW}^{*}.$$

Solving for $\frac{{I}_{NW}^{*}}{N}={P}_{NW}^{*}$ and $\frac{{I}_{RW}^{*}}{N}={P}_{RW}^{*}$ yields:

22 $${P}_{NW}^{*}=\frac{{R}_{NW}{P}_{W}^{*} \left(1-{f}_{R}\right)}{1+{R}_{NW}{P}_{W}^{*}},$$

23 $${P}_{RW}^{*}=\frac{{R}_{RW}{P}_{W}^{*}{f}_{R}}{1+{R}_{RW}{P}_{W}^{*}}.$$

where ${P}^{*}={P}_{NW}^{*}+{P}_{RW}^{*}$. We can solve these two equations with two unknowns to get expressions for the prevalence as function of the reproduction ratios and ${f}_{R}$:

24 $$P_{NW}^* = \frac{{\left( {1 - {f_R}} \right)\left( { - {R_{RW}} + {R_{NW}}\left( { - 1 + {R_{RW}} + {R_{RW}}\sqrt {\frac{1}{{R_{NW}^2}} + \frac{{ - 2 + 4{f_R} - \frac{2}{{{R_{RW}}}}}}{{{R_{NW}}}} + \frac{{1 - 2{R_{RW}} - 4{f_R}{R_{RW}} + R_{RW}^2}}{{R_{RW}^2}}} } \right)} \right)}}{{{R_{RW}} + {R_{NW}} - \left( {1 + {R_{RW}} + {R_{RW}}\sqrt {\frac{1}{{R_{NW}^2}} + \frac{{ - 2 + 4{f_R} - \frac{2}{{{R_{RW}}}}}}{{{R_{NW}}}} + \frac{{1 - 2{R_{RW}} - 4{f_R}{R_{RW}} + R_{RW}^2}}{{R_{RW}^2}}} } \right)}},$$

25 $$P_{RW}^* = \frac{{{f_R}\left( { - {R_{RW}} + {R_{NW}}\left( { - 1 + {R_{RW}} + {R_{RW}}\sqrt {\frac{1}{{R_{NW}^2}} + \frac{{ - 2 + 4{f_R} - \frac{2}{{{R_{RW}}}}}}{{{R_{NW}}}} + \frac{{1 - 2{R_{RW}} - 4{f_R}{R_{RW}} + R_{RW}^2}}{{R_{RW}^2}}} } \right)} \right)}}{{ - {R_{RW}} + {R_{NW}}\left( {1 + {R_{RW}} + {R_{RW}}\sqrt {\frac{1}{{R_{NW}^2}} + \frac{{ - 2 + 4{f_R} - \frac{2}{{{R_{RW}}}}}}{{{R_{NW}}}} + \frac{{1 - 2{R_{RW}} - 4{f_R}{R_{RW}} + R_{RW}^2}}{{R_{RW}^2}}} } \right)}},$$

26 $${P}_{W}^{*}=\frac{1}{2}\left(-\frac{1}{{R}_{NW}}+\frac{-1+{R}_{RW}+{R}_{RW}\sqrt{\frac{1}{{R}_{NW}^{2}}+\frac{-2+4{f}_{R}- \frac{2}{{R}_{RW}}}{{R}_{NW}}+\frac{1-2{R}_{RW}-4{f}_{R}{R}_{RW}+{R}_{RW}^{2}}{{R}_{RW}^{2}}}}{{R}_{RW}}\right).$$
